# Supplementary material for: Adult mouse dorsal root ganglia neurons form aberrant glutamatergic connections in dissociated cultures
Source: PLoS One. 2021 Mar 3;16(3):e0246924. doi: 10.1371/journal.pone.0246924 (PMC7928449; doi:10.1371/journal.pone.0246924)
Supplement: S1 File — (DOCX) [file pone.0246924.s001.docx]

***S1 File. Solutions and media***

***Dissection Medium:*** Roswell Park Memorial Institute, (RPMI, Sigma), medium with 1mM GlutaMAX-I (Gibco) supplement and 1% antibiotic-antimycotic containing (100U penicillin, 100mg streptomycin, 250ng/mL amphotericin-B, Sigma).

***Culture Medium:*** Used for culture and maintenance of the cells consisting of Neurobasal-A (Gibco) supplemented with 2% B27 (Gibco), 2mM GlutaMAX-I and 1% antibiotic solution.

***Enzyme Solution 1****:* 100U/mL collagenase (Sigma) enzyme added to culture medium.

***Enzyme Solution 2:*** 1mg/mL trypsin solution (Sigma) and 50µg/mL DNase (Sigma) enzyme added to culture medium.

***Enzyme Inhibition Medium:*** Culture medium including 700µg/mL trypsin inhibitor (Sigma) and 10% fetal bovine serum (FBS, Sigma).

***Gradient Preparation*:** Percoll (Sigma) solution was diluted at 10, 35 and 60% concentrations in culture medium, to prepare a three-layer purification-gradient. Where neurons mainly aggregate at the middle layer after centrifuge.

***Antagonists:*** NMDA antagonist AP5 (Sigma) and AMPA-Kainate antagonist CNQX (Sigma) were used 100µM and 10µM respectively and incubated in 37º, 5% CO_2_ incubator for 30 minutes prior to the experiments.

***ICC Solutions:*** 3% bovine serum albumin (BSA, Sigma), 1% goat serum (Sigma) and 0.3% sodium azide (Sigma) diluted in phosphate buffer saline (PBS, Sigma) and 3% BSA, 1% goat serum and 0.1% Triton-X (Sigma) diluted in PBS were used as the blocking and the dilution solutions respectively.
